# Annealing-Induced Off-Stoichiometric and Structural Alterations in Ca^2+^- and Y^3+^-Stabilized Zirconia Ceramics

**DOI:** 10.3390/ma14195555

**Published:** 2021-09-24

**Authors:** Wenliang Zhu, Shizuka Nakashima, Elia Marin, Hui Gu, Giuseppe Pezzotti

**Affiliations:** 1 Ceramic Physics Laboratory, Kyoto Institute of Technology, Sakyo-ku, Matsugasaki, Kyoto 606-8585, Japan; b5112096@edu.kit.ac.jp (S.N.); elia-marin@kit.ac.jp (E.M.); pezzotti@kit.ac.jp (G.P.); 2 School of Materials Science and Engineering, Shanghai University, Shanghai 200444, China

**Keywords:** ceramics, phase transformation, Raman spectroscopy, thermodynamic stability, stabilized zirconia polycrystal

## Abstract

In the current study, high-temperature stability was investigated in two types of zirconia ceramics stabilized with two different additives, namely, calcia and yttria. The evolutions of structure and oxygen-vacancy-related defects upon annealing in air were investigated as a function of temperature by combining X-ray diffractometry with Raman, X-ray photoelectron and cathodoluminescence spectroscopies. We systematically characterized variations in the concentration of oxygen vacancies and hydroxyl groups during thermal treatments and linked them to structural alterations and polymorphic transformation. With this approach, we clarified how the combined effects of different dopants and temperature impacted on structural development and on the thermal stability of the oxygen-vacancy-related defect complex.

## 1. Introduction

Stabilized zirconia (ZrO_2_) ceramics possess excellent properties, such as high chemical stability and strong thermal shock resistance, as well as biocompatibility, and they can be used in a variety of applications, including structural components, damage/corrosion-resistant coatings, oxygen sensors and dental implantation [1,2,3]. In addition, high corrosion resistance, high ionic conductivity and low thermal conductivity make these ceramics attractive for applications as electrolyte membrane and as components in composite cathodes for solid oxide fuel cells (SOFCs) [4,5].

In general, the stabilization of cubic/tetragonal zirconia ceramics can be achieved by adding hetero-valent ions (most commonly Y^3+^), as these dopants introduce oxygen vacancies in the zirconia lattice that modify the crystallographic and electronic structures. Modifications in vacancy concentration and changes in electronic structures may be used in tailoring the transport, chemical and optical properties of doped zirconia [6], making them attractive for applications in radiation- or plasma-induced surface catalysis [7].

However, defect alteration and phase instability of zirconia in specific service environments (e.g., high temperature and humidity) may cause a degradation of these properties, even if the zirconia ceramics are fully stabilized [8]. For example, 8–9 mol% Y-stabilized zirconia (YSZ) for SOFC applications appears to be situated in the two-phase field (cubic + tetragonal) of the phase diagram at working temperatures, but coarsening of the tetragonal regions and decomposition on the cationic sublattice into Y-enriched and depleted regions on the nanoscale may induce electrical degradation (i.e., a lowered ionic conductivity) during operation [9]. Such undesirable environmentally dependent polymorphism in zirconia ceramics depends on type, fraction and distribution of dopants; grain size; grain boundary composition; and process control [10,11,12,13,14]. 

In engineering practice, it is well known that the performance of stabilized zirconia at an elevated temperature strongly depends on both the type and content of oxygen-vacancy-related defects existing in the material; e.g., it has been reported that the annealing of stabilized zirconia in air or vacuum could change the phase composition and cause some redistribution of the stabilizing oxide as well as altering the fracture toughness of the material [15,16]; however, defect variations in stabilized zirconia induced by thermal processes are rarely characterized and reported. Recently, we have clarified some new aspects of YSZ zirconia polymorphic transformation in different environments as a function of dopant content [17,18]. Oxygen vacancies and surface/lattice hydroxyl defects were found to play critical roles in distinct off-stoichiometric phenomena, including hydroxylation, hydroxyl migration, surface reconstruction and dehydroxylation during hydrothermal/thermal treatments. 

In this paper, a comparison is made between two types of zirconia ceramics stabilized with different dopants (calcia and yttria), whose cations have different valence states. The main purpose is to investigate the thermal stability of the structures and the evolution of oxygen-vacancy-related defects upon thermal treatment. The defect variation in response to annealing in air at different temperatures is analyzed using cathodoluminescence spectroscopy, and structural changes are investigated using X-ray diffraction analysis as well as Raman and X-ray photoelectron spectroscopies. The results reveal a strong dependence of the zirconia structure and lattice defects on both annealing temperature and type of dopant.

## 2. Materials and Methods

Two different series of commercially available zirconia samples were investigated in this research. They were mainly stabilized by 8 mol% yttria (and a small amount of calcia) and 4.2 mol% calcia (AS ONE Corporation, Osaka, Japan) (henceforth simply referred to as YSZ and CaSZ, respectively). The samples also contained small amounts of silica and alumina as sintering additives (<2%).

Samples were cut into a number of pieces, and some of these pieces were subjected to isothermal treatments in a conventional electric heater (NHK-120H-II, Nitto Kagaku Co. LTD., Nagoya, Japan) operating at 300, 500 or 700 °C in air for 12 h. 

Raman spectroscopic analyses were performed at room temperature (RT) before and after thermal treatments using a single monochromator (T-64000, Jobin-Ivon/Horiba Group, Kyoto, Japan). Raman maps 50 × 50 μm^2^ in size were constructed with a step of 10 μm to obtain average spectra.

X-ray photoelectron spectroscopy (XPS) experiments were performed on the top surfaces of these samples using a photoelectron spectrometer (JPS-9010 MC; JEOL Ltd., Tokyo, Japan). The monochromatic MgKα line was used as the X-ray source (output 10 kV, 10 mA). 

X-ray diffraction (XRD) analyses of the YSZ and CaSZ samples were performed on a Rigaku Miniflex 600 system (Rigaku Corporation, Tokyo, Japan), using CuKα radiation (40 kV and 15 mA). 

Cathodoluminescence (CL) analyses were carried out in a field-emission gun scanning electron microscope (FEG-SEM, SE-4300, Hitachi Co., Tokyo, Japan). The microscope was equipped with a CL device consisting of an ellipsoidal mirror and a bundle of optical fibers that were respectively used to collect and focus the electron-stimulated luminescence emitted by the sample into a high, spectrally resolved light monochromator (Triax 320, Jobin-Yvon/Horiba Group, Tokyo, Japan). In performing CL measurements, the exact same experimental conditions were applied to avoid introducing additional variables, and respective average spectra were obtained by averaging the spectra collected at 10 different locations. The acceleration voltage and the beam current were fixed in all experiments at 5 kV and 180 pA, respectively. 

## 3. Results

### 3.1. X-ray Diffraction

**Figure 1:**
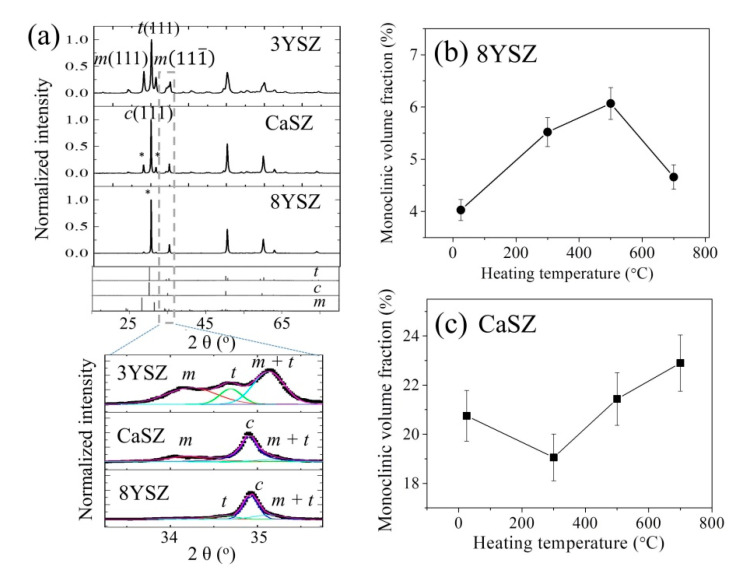
(**a**) XRD patterns for the samples of YSZ (8 mol%), CaSZ and 3 mol% YSZ, with an enlargement of the 2θ peaks in the range from 33° to 36° shown in the bottom inset, and variations of monoclinic volume fraction *V_m_* of the samples with annealing temperature for (**b**) YSZ and (**c**) CaSZ.

Figure 1a shows the X-ray diffraction patterns collected on YSZ and CaSZ samples. For comparison, the spectrum of a 3 mol% yttria partially stabilized zirconia that consisted of both tetragonal and monoclinic phases is also shown in the figure. As can be seen, the sample of 8YSZ shows mainly diffraction peaks characteristic of cubic zirconia together with two rather weak peaks belonging to the monoclinic polymorph (located at about 28.2 and 31.4° for 2θ). In the case of CaSZ, the intensity of monoclinic phase-related peaks markedly increased, despite the dominating presence of cubic zirconia. An enlargement of the peaks in the range from 33 to 36° clearly confirmed the observed phenomena, also revealing the presence of an almost negligible amount of the tetragonal phase in the two high-doping samples (cf. peculiar bands of these phases labeled in the inset as m, t and c). 

After spectral deconvolution using Lorentzian functions, the monoclinic volume fraction of zirconia in the samples could be calculated according to the peaks in the range from 33° to 36° (marked with *; cf. bottom inset in Figure 1a) [19].

(1) $$V_{m}=\frac{I_{m}\left(11\bar{1}\right)+I_{m}\left(111\right)}{I_{m}\left(11\bar{1}\right)+I_{m}\left(111\right)+I_{t,c}\left(111\right)}$$

The calculated results for samples subjected to thermal treatments at different temperatures are shown in Figure 1b,c for YSZ and CaSZ, respectively. With increasing temperature, the monoclinic fraction slightly increased until 500 °C but then decreased at 700 °C for YSZ, while in the case of CaSZ, it initially decreased at 300 °C and then increased with a further increase in temperature. These different trends indicate a different modification of defective clusters in zirconia as a function of annealing temperature for the cubic-based samples. 

### 3.2. Raman Spectroscopy

**Figure 2:**
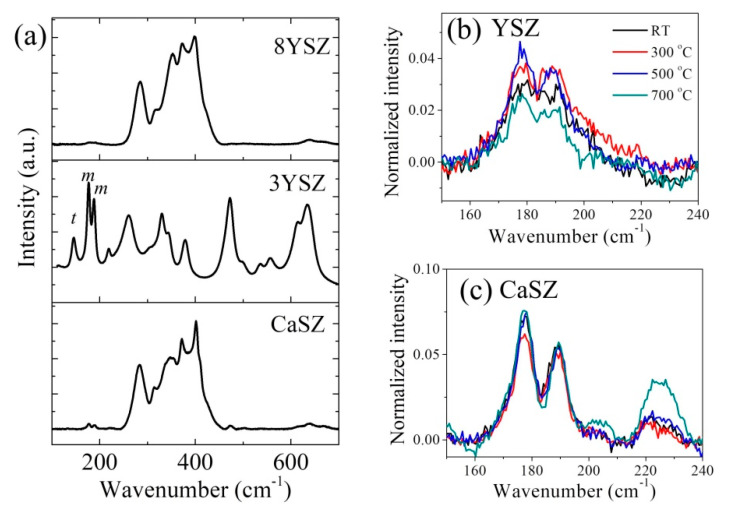
(**a**) Typical Raman spectra of the samples of YSZ (8 mol%), CaSZ and 3 mol% YSZ, and variations of Raman spectra in the range from 150 to 240 cm^−1^ with annealing temperatures for (**b**) YSZ and (**c**) CaSZ.

Figure 2a shows a comparison between Raman spectra of untreated YSZ and CaSZ samples and the reference 3YSZ sample. Unlike the reference 3YSZ sample (central spectrum), which showed only bands from both monoclinic and tetragonal polymorphs (cf. labels m and t, respectively), both 8YSZ and CaSZ samples mainly displayed strong bands in the wavenumber range 250–420 cm^−1^ besides very weak signals from the monoclinic polymorph at 177 and 188 cm^−1^. The 8YSZ and CaSZ spectra recorded in this study are also different from what we previously reported for 8 mol% yttria-doped zirconia, which showed a strong and broad band at 610 cm^−1^ belonging to cubic phase [18]. The bands observed between 250 and 420 cm^−1^ can be attributed to mixed Zr-O-M vibrations (M = Zr, Ca, Y, Al and Si) [20,21]. The rationale for this assignment resides in the fact that heavy doping by hetero-ions with largely different atomic radii causes a strong local lattice distortion, which breaks the symmetry of the cubic phase and leads to the appearance of otherwise inactive Raman modes in this spectral range. In addition, in the case of YSZ sintered at a relatively high temperature (e.g., 1450–1600 °C) and containing divalent ions, a dual cubic–tetragonal microstructure may occur [22]. Additionally, the presence of a small amount of tetragonal and monoclinic phases due to an inhomogeneous distribution of the hetero-ions in the zirconia lattice can make a contribution to the above-mentioned spectral range. An important implication of the present Raman data is that lattice imperfections could be clearly revealed as a consequence of the difference in the Raman scattering cross-section between tetragonal and cubic phases and of the higher sensitivity of the Raman probe as compared with XRD.

Upon increasing the temperature of the thermal treatments, the YSZ samples showed a gradual increase in the intensity of monoclinic peaks with respect to that of the 270 cm^−1^ band, followed by a decrease at 700 °C (cf. Figure 2b). On the other hand, the CaSZ sample exhibited a slight decrease at 300 °C and then an increase upon further increasing the temperature (cf. Figure 2c). 

### 3.3. XPS Spectroscopy

**Figure 3:**
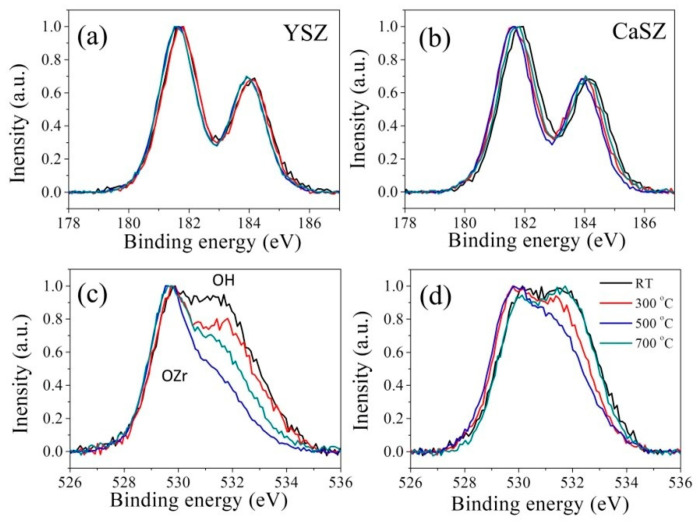
Variations of (**a**,**b**) Zr3d and (**c**,**d**) O1s XP spectra with thermal treatment temperature for the (**a**,**c**) YSZ and (**b**,**d**) CaSZ samples.

In order to further investigate the thermal stability of zirconia upon doping with yttria or calcia, XPS experiments were carried out on YSZ and CaSZ samples, as the variations of Zr3d, and O1s XP spectra for thermally treated samples, as shown in Figure 3. The two peaks in the Zr3d XP spectra (Figure 3a,b) originate from Zr3d_5/2_ (~182 eV) and Zr3d_3/2_ (~184 eV) in Zr-O bonds, while O-Zr (~530 eV) and O-H (~532 eV) contributed the broad peaks in the O1s spectra (Figure 3c,d). The Zr3d and O1s XP peaks of the samples consisted of contributions from both Zr-O binding and O-Zr/O-H clusters in all zirconia phases. 

A negligible change in the Zr3d spectral morphology with temperature could be found for YSZ, while CaSZ exhibited a shift of the peak position to lower binding energies when the temperature was increased to 500 °C and then a shift back at 700 °C. In the case of O1s XP spectra, despite a similar trend in the variations observed for both samples, the fraction of the OH-related peak decreased at a much faster rate in YSZ than in CaSZ in response to increases in temperature of up to 500 °C, and then it increased again at 700 °C. Indeed, the sample thermally treated at 700 °C showed a spectrum almost the same as that of the untreated sample for CaSZ.

### 3.4. CL Spectroscopy

**Figure 4:**
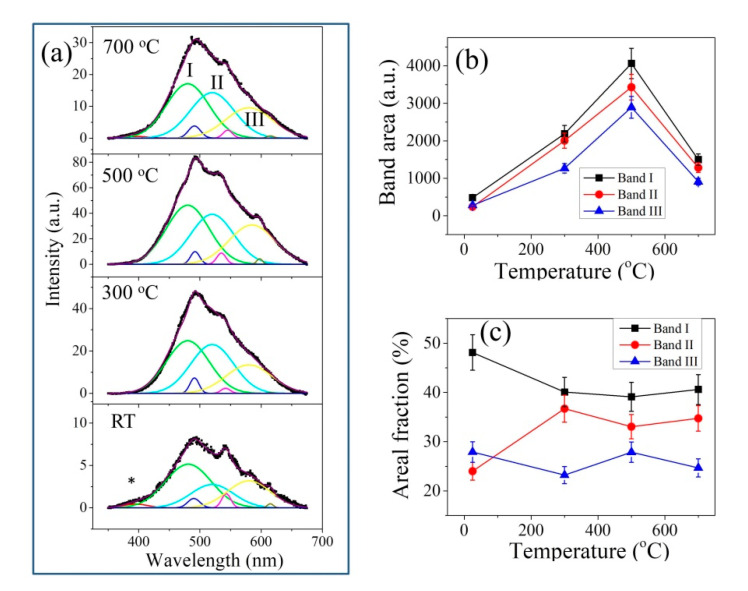
Variations of (**a**) CL spectra, (**b**) band area and (**c**) areal percent of the sub-bands with annealing temperature for the YSZ samples.

**Figure 5:**
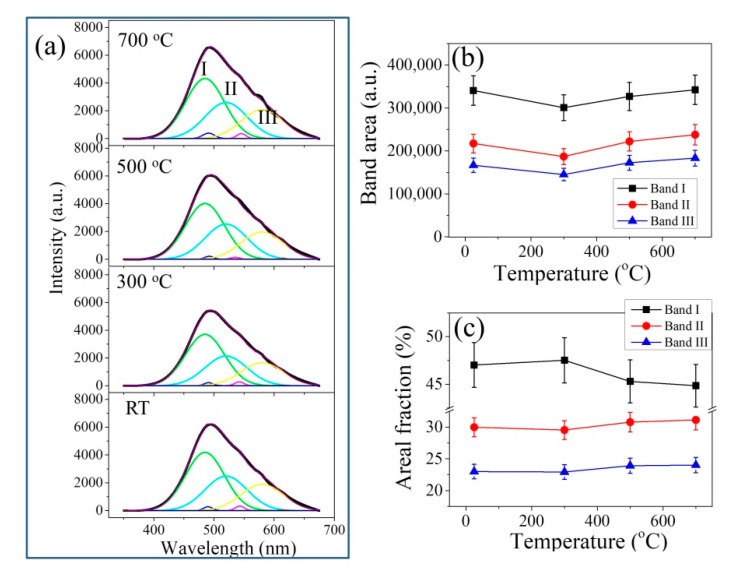
Variations of (**a**) CL spectra, (**b**) band area and (**c**) areal percent of the sub-bands with annealing temperature for the CaSZ samples.

Figure 4 and Figure 5 show the average CL spectra of the investigated thermally treated samples. All spectra show a main band with its maximum located at around 490 nm and shoulder sub-bands on both sides, especially in the higher wavelength region. These bands arose from luminescent defects in the zirconia lattice and were also observed when zirconia was irradiated by a laser with energy far lower than the band gap of zirconia (>5 eV) [23,24,25]. 

Spectral deconvolution of the CL spectra was performed based on previous studies of yttria-doped zirconia, which showed the presence of three sub-bands (denoted as Bands I–III, cf. Figure 4a and Figure 5a) contributed by oxygen-vacancy-related defect complexes F^+^, F^+^_A_ and F^+^_AA_ centers [25,26]. The F^+^ center is associated with a cluster of singly occupied oxygen vacancy in the neighborhood of two zirconium ions (i.e., Zr-V_o_^•^-Zr), which catches one electron to become a positive charge center. The F^+^_A_ and F^+^_AA_ centers are associated with defective oxygen vacancy clusters neighboring one and two substituted hetero-ions/impurity ions, respectively (e.g., in the case of YSZ, Y-V_o_-Zr and Y-V_o_-Y). Besides these strong bands, a band at around 693 nm; a weak peak at around 390 nm; and some weak bands located at around 500, 540 and 610 nm could also be observed, originating from Cr impurity (R-lines), oxygen vacancy (F-center) in alumina (* in Figure 4a) and oxygen-vacancy-related defects in silica, respectively [27,28].

As shown in Figure 4b, upon thermal treatment of YSZ, the intensity of the overlapping broad signal first increased up to 500 °C and then decreased at a higher temperature. In addition, Band I first showed a decrease at 500 °C but barely changed with further increasing temperature; Band II showed a nearly opposite trend with respect to Band I, while no significant variations were found for Band III (Figure 4c).

However, in the case of CaSZ, which showed much stronger signals than YSZ, only a slight change in spectral intensity could be observed with increasing temperature (Figure 5b). After spectral deconvolution, a slight increase in Band I could be found at 300 °C and then a decrease with increasing temperature. Band II showed an almost negligible trend (within the error) opposite to Band I (Figure 5c).

Finally, it is worth noting that the oxygen-vacancy-related band in alumina almost disappeared after thermal treatment (cf. * in Figure 4a), indicating an incorporation of oxygen in the lattice during thermal treatments.

## 4. Discussion

In our previous papers [17,29], we have noticed a significant difference in the kinetics of polymorphic transformation of zirconia in different environments, and the critical roles played by oxygen vacancy and surface/lattice hydroxyl defects have been discussed due to the occurrence of distinct off-stoichiometric chemical effects. These effects included hydroxylation, dehydroxylation hydroxyl migration and surface reconstruction during treatments.

As generally recognized, the polymorphic stability of zirconia is associated with the presence of oxygen vacancies in the lattice. Upon adding yttria or calcia in zirconia powders during the sintering process, the trivalent yttrium (Y^3+^) or divalent calcium (Ca^2+^) ions may substitute for zirconium (Zr^4+^) in the zirconia lattice with the formation of negatively charged defects, Y_Zr_′ and Ca_Zr_″, which results in the generation of oxygen vacancies for charge compensation. The oxygen vacancies associated with the “relaxed” lattice stabilize the tetragonal/cubic polymorph at room temperature, avoiding spontaneous collapse into a monoclinic configuration [30]. Destabilization of the tetragonal zirconia phase is induced by the reduction in oxygen vacancy concentration, which promotes its transformation into the monoclinic polymorph [31].

After sample fabrication, if samples are exposed to humid environments at low temperatures, surface hydroxylation may occur due to the reaction of the ceramic surface with adsorbed water. Hydroxylation leads to the formation of hydroxyl binding to cations at the very surface of the cubic zirconia phase or to the incorporation of H_2_O molecules into the ZrO_2_ lattice [17]:

(2) $$\mathrm{Zr}\left(Y,\mathrm{Ca}\right)O_{2}+{\;H}_{2}O\;\to \mathrm{Zr}\left(Y,\mathrm{Ca}\right)O{\left(\mathrm{OH}\right)}_{2}$$

(3) $${\;H}_{2}O\;+{\;V}_{ö}+{\;O}_{O}^{x}\to 2{\left(\mathrm{OH}\right)}_{O}^{•}$$

This kind of destabilization of the tetragonal polymorph, as induced by the reduction in V_O_ in biological or hydrothermal environments, is a very common phenomenon in 3Y-TZP, known as low-temperature aging [13,17].

Indeed, in the present cases of YSZ and CaSZ, the presence of a significant amount of Zr-OH on the surface of the untreated samples due to environmental humidity could be confirmed by XPS analysis (cf. Figure 3). The incorporation of hydroxyl group in the lattice can be on the free surface (OH)^s^ (Equation (2)) or in the sub-lattice as the defective hydroxyl proton ${\left(\mathrm{OH}\right)}_{O}^{•}$ (Equation (3)), resulting in the coexistence of two distinct types of surface hydroxyl groups in the materials [32].

As has been reported, increasing yttria content could increase the ionic conductivity of YSZ, showing a maximum at around 8–9 mol%, which is almost temperature independent [33]. Upon thermal treatment, the high oxygen ion conductivity in the presence of oxygen vacancies allows oxygen ions to hop from vacancy to vacancy in the lattice of yttria stabilized zirconia [34], and the generated surface hydroxyl may migrate in the depth direction to fill the oxygen vacancy in the bulk lattice V_o_^b^ followed by surface reconstruction [35].

(4) $${\;V}_{O}^{b}+{\left(\mathrm{OH}\right)}^{s}\;\to {\left(\mathrm{OH}\right)}_{O}^{b}$$

(5) $${\;V}_{O}^{b}+{\left(\mathrm{OH}\right)}_{O}^{s}\to {\left(\mathrm{OH}\right)}_{O}^{b}+V_{O}^{s}$$

Note that reactions (2)–(5) are reversible and strongly dependent on temperature, concentration/pressure of water in air and contents of both oxygen vacancies and hydroxyls in the zirconia lattice, in addition to being affected by the stability of oxygen-vacancy-related defect complexes.

Accordingly, when the sample is subjected to thermal treatments with sufficient thermal energy, dehydroxylation can occur, which removes the surface/lattice hydroxyl defects [29]:

(6) $$2{\left(\mathrm{OH}\right)}_{O}^{•}\to H_{2}O\;+{\;V}_{ö}+{\;O}_{O}^{x}$$

In other words, migration of OH from the sample depth toward its surface, followed by the formation of water, can also take place (inverse reactions of Reactions (2), (4) and (5)). Indeed, dehydroxylation of the protonated hydroxyl moiety can easily take place in alumina when exposed to a hydrothermal environment [36]. This process results in a gradual enhancement of the V^+^_O_ sites (i.e., oxygen vacancy capturing one electron), which is observed in an increase in the CL F^+^ band with increasing autoclaving time [10].

In the current cases of CaSZ and 8YSZ, as mentioned above, the formation of an oxygen vacancy to compensate the defects of Ca_Zr_″ and Y_Zr_′ for electrical neutrality may result in the generation of different kinds of defect combinations: <i> two isolated defects, [Ca_Zr_″-O……Zr-$V_{ö}$] (or [Y_Zr_′-O……Zr-$V_{ö}$] for 8YSZ), with long-distance charge compensation; <ii> an isolated defect complex, [Ca_Zr_″-$V_{ö}$] (or [Y_Zr_′-$V_{ö}$]); and <iii> a defect complex, [Ca_Zr_″-$V_{ö}$] ([Y_Zr_′-$V_{ö}$]), neighboring another Ca_Zr_″ (Y_Zr_′) defect, [Ca_Zr_″-$V_{ö}$…Ca_Zr_″-O] (or [Y_Zr_′-$V_{ö}$-Y_Zr_′]). Since the charges of Ca_Zr_″ and $V_{ö}$ are equivalent, the neutral defect complex [Ca_Zr_″-$V_{ö}$] is much more stable than the positively charged [Y_Zr_′-$V_{ö}$]. That is, the charge balance makes the defect complexes of [Ca_Zr_″-$V_{ö}$] and [Y_Zr_′-$V_{ö}$-Y_Zr_′] stable so that oxygen vacancies are deactivated as promoters of ion migration to accommodate hydroxyl protons at low temperatures.

Moreover, similar to the case of 3Y-TZP, and as a consequence of the low content of added Ca and Y, the distribution of Ca and Y atoms is not uniform: each cluster of 25 crystal unit cells contains and shares only 4.2 Ca atoms for CaSZ (~8Y + *x*Ca for 8YSZ) and 4.2 V_O_ (> 4 V_O_). Most unit cells do not contain Ca (Y) and V_O_, and their cubic structures are maintained at room temperature according to the constraint operated by Ca(Y)-stabilized unit cells away from them. As a result, regions with lower Ca(Y) concentration are more prone to phase transformation, with a higher monoclinic fraction being observed in the untreated CaSZ sample (cf. Figure 1b,c), which has a smaller content of cation ion than 8YSZ.

Note that the presence of oxygen vacancy and hetero-ions in zirconia results in a local distortion of the surrounding/neighboring lattice cells, which may form a quasi-continuum of defect energy states in the band gap due to the different distances to oxygen vacancy [23]. Excitation by electrons leads to the formation of electron–hole pairs, which can be trapped in different meta-stable energy states within the band gap. When an electron transits from the excited state to the ground state, a photon will be emitted corresponding to the wavelength of the observed bands. Since the non-degenerate monoclinic phase shows a significant alteration of oxygen surrounding the Zr sites (reduced coordination number of Zr from 8 to 7 and increased average O–O distance) [37], the quasi-continuum of defective energy states can be formed more easily, and it is assumed to exhibit much higher luminescence efficiency than the degenerate stabilized phases (tetragonal and cubic). Such a hypothesis of higher luminescence efficiency for the non-degenerate monoclinic phase is supported by the experimental observation of enhanced CL intensities and gradually increased monoclinic fraction with aging time for 3Y-TZP in autoclave, despite a decrease in oxygen vacancy because of the formation of the defective hydroxyl proton ${\left(\mathrm{OH}\right)}_{O}^{•}$ [10,29]. Another example of support comes from a recent report stating that with increasing yttria content in YSZ, the luminescence was gradually inhibited, along with a decrease in monoclinic fraction and an increase in tetragonal/cubic fraction [26]. However, it should be noted that for band-to-band excitation, reduced electron–hole recombination could also contribute to the inhibition of luminescence due to the capture of electrons and holes by oxygen vacancies and hetero-ions, respectively.

Accordingly, the observed higher monoclinic fraction in CaSZ (cf. Figure 1b,c) might be responsible for the observed stronger luminescence than that of 8YSZ (cf. Figure 4a and Figure 5a).

Upon thermal treatment at 300 °C for CaSZ, surface dehydroxylation and minor migration of surface hydroxyl to the depth to fill V_o_ in the isolated defects [Ca_Zr_″-O……Zr-$V_{ö}$] occurred, which resulted in a slight decrease in surface OH (cf. Figure 3d). As surface oxygen vacancies are generated by dehydroxylation, similar to the cases of thermally treated YSZ with high initial *V_m_* [18], polymorphic transformation from the monoclinic to the tetragonal phase could be expected (cf. Figure 1c and Figure 2c). Because of the formation of $V_{ö}$ and of the decreased monoclinic fraction, a decrease in the intensity of the overall CL band could be observed. Moreover, the higher fraction of the Zr-$V_{ö}$-Zr-related Band I indicated the formation of unconstrained oxygen vacancies on the surface. However, at higher temperatures (≥500 °C), the oxygen vacancies in the defect complex [Ca_Zr_″-$V_{ö}$] can be thermally activated for ion conduction, since a higher thermal energy may allow oxygen ions to hop from the surface toward oxygen vacancies in the depth through the defect complex. Bulk oxygen vacancies might be reduced by the migration of surface oxygens and hydroxyls, although surface dehydroxylation may still contribute to the formation of oxygen vacancies at the material surface. Therefore, a reduction in oxygen vacancy resulted in an increase in the monoclinic phase (cf. Figure 1c and Figure 2c), as well as in the observed increase in CL band intensity (cf. Figure 5a) at higher temperatures. Moreover, as oxygen vacancies begin to move under the coulombic attraction between two oppositely charged defects, it is easier for V_O_ in the isolated defects [Ca_Zr_″-O……Zr-$V_{ö}$] to approach the cation defect Ca_Zr_″ and to form the defect complex [Ca_Zr_″-$V_{ö}$]. Accordingly, the fraction of Band I decreased upon annealing at higher temperatures (Figure 5c). Note that at 700 °C, the content of surface hydroxyls returned to high values (cf. Figure 3d). This can be associated with the initiation of inverse reactions in Equations (4) and (5), as hydroxyls in the bulk started to migrate to the surface for dehydroxylation, although the reactions in Equations (4) and (5) were still dominant.

In regard to YSZ, upon thermal treatment at lower temperatures, the occurrence of surface dehydroxylation and surface hydroxyl migration to the depth to fill V_o_ in the isolated defects [Y_Zr_′-O……Zr-$V_{ö}$] resulted in a decrease in surface OH (cf. Figure 3c). Oxygen vacancies in the defect complex [Y_Zr_′-$V_{ö}$] were activated for the migration of surface oxygens and hydroxyls, which reduced the content of bulk oxygen vacancies and resulted in a gradual increase in the monoclinic fraction (cf. Figure 1b and Figure 2b) and in the CL band intensity (cf. Figure 4b). Moreover, the mobility of oxygen vacancy and the coulombic attraction made the isolated V_O_ in the defects [Y_Zr_′-O……Zr-$V_{ö}$] approached the cation defect Y_Zr_′ to form the defect complex [Y_Zr_′-$V_{ö}$]. Accordingly, the fraction of Band II increased while that of Band I decreased with an increasing annealing temperature (cf. Figure 4c). Since the defective cluster [Y_Zr_′-$V_{ö}$-Y_Zr_′] is relatively stable, the fraction of Band III showed a minor change with temperature.

Similarly, the high content of surface hydroxyl at 700 °C (cf. Figure 3c) might be associated with the occurrence of inverse reactions in Equations (4) and (5), as the hydroxyl in the bulk migrated to the surface for dehydroxylation. The generation of oxygen vacancies in the bulk might cause transformation from the monoclinic to the tetragonal phase at high temperatures (cf. Figure 1b and Figure 2b) [18]. Thus, a decrease was seen in the overall CL band intensity (Figure 4). Moreover, the small change in the fraction of the Zr-$V_{ö}$-Zr-related Band I indicated the formation of both constrained and unconstrained oxygen vacancies at the surface due to the relatively weak coulombic attraction between Y_Zr_′ and $V_{ö}$.

As mentioned above, for stabilized zirconia ceramics, the defect alteration and phase instability of zirconia in specific service environments may have a marked influence on their properties, and the clarification of annealing-induced off-stoichiometric and structural alterations is expected to help in fabricating better devices for high-temperature applications.

## 5. Conclusions

In this study, the thermal stability of structure and oxygen-vacancy-related defects in response to annealing in air at different temperatures were investigated in two zirconia ceramics stabilized with two different additives, namely, calcia and yttria. According to the analyses of structure and defects using XRD, Raman, XPS and CL spectroscopies, it was determined that the alteration in structure accompanying phase transformation in zirconia was related to the variation in oxygen vacancies and hydroxyl groups during thermal treatments. In addition, the thermal stability of the oxygen-vacancy-related defect complex, as well as the microstructure, showed a dependence on both the type of additive and annealing temperature. As the presence of oxygen vacancies affects various properties of stabilized zirconia, determining the behavior of oxygen vacancies with respect to annealing is expected to help in fabricating better devices for high-temperature applications.

## Data Availability

No new data were created or analyzed in this study. Data sharing is not applicable to this article.
